# Incidence, survival, and prevalence trends for myocardial infarction, stroke, hip fracture, and cancer across Swedish birth cohorts: a population-based register study

**DOI:** 10.1016/j.lanepe.2026.101785

**Published:** 2026-07-21

**Authors:** Anna-Kathleen Piereth, Katharina Schmidt-Mende, Karin Modig, Marcus Ebeling

**Affiliations:** a Max Planck Institute for Demographic Research, Konrad-Zuse-Str. 1, 18057, Rostock, Germany; b Karolinska Institutet, Institute of Environmental Medicine, Unit of Epidemiology, Nobels väg 13, Solna, 171 77, Stockholm, Sweden; c Academic Primary Health Care Centre, Stockholm Region, Solnavägen 1E (Torsplan), 113 65, Stockholm, Sweden; d Karolinska Institutet, Department of Neurobiology, Care Sciences and Society, Division of Family Medicine and Primary Care, Alfred Nobels allé 23, A4, 141 83, Huddinge, Sweden; e Max Planck – University of Helsinki Center for Social Inequalities in Population Health (MaxHel Center), Rostock, Germany

**Keywords:** Disease prevention, Incidence, Survival, Prevalence, Major diseases, Cohort-to-cohort change

## Abstract

**Background:**

Medical progress reshapes population health through preventing disease onset and improving disease survival. It is unclear which of these forces has advanced and altered the disease landscape more. This study compares age-specific changes in incidence and survival and their influence on prevalence across major diseases.

**Methods:**

Using register data covering the total Swedish population from 1994 to 2022 and the birth cohorts 1904 to 1960, we traced first events of myocardial infarction (MI), stroke, hip fracture, and cancer. We analysed incidence rates, one- and five-year survival for ages 60–90. We compared average cohort-to-cohort changes in all measures, and their effect on prevalence at ages 70, 80, and 90 by cohort and sex.

**Findings:**

For MI, stroke, and hip fracture, relative improvements in incidence outpaced those in survival at every age. For example, MI incidence among men aged 60 declined on average by 2.0% (SD = 7.0%) every year, resulting in an absolute decrease from 7 (274/37,411; cohort 1934) to 4 (234/54,144; cohort 1960) cases per 1,000 person-years. One-year survival for the same group increased on average by 0.9% (SD = 3.2%) yearly, leading to an absolute increase from 70% (193/274) to 88% (206/234). Prevalence declined for MI, stroke, and hip fracture with the magnitude varying by age group. The largest decrease occurred for MI among women aged 80, where the prevalence declined from 4.5% (1,554 incident cases per 34,676 persons alive; cohort 1924) to 2.7% (1,105/41,122; cohort 1942), corresponding to a relative decrease of −40% during the observation period. Cancer showed increasing incidence, improvements in survival, and increases in prevalence. The largest prevalence increase was observed among women aged 80, where prevalence rose from 8.0% (2,832/35,568; cohort 1922) to 12.5% (4,563/36,609; cohort 1941), corresponding to a relative increase of 57%.

**Interpretation:**

Progress against MI, stroke, and hip fracture was strongest before diagnosis (primary prevention), but for cancer, especially evident at or after diagnosis (secondary and tertiary prevention). This pattern reflects a shift in the disease landscape of the Swedish population from severe acute events to chronic conditions.

**Funding:**

Not applicable.

**Research in context**

**Evidence before this study**

We conducted a systematic search of the PubMed and Web of Science databases from their inception to 30 November 2025. Limited to terms in the title or abstract, we used search terms related to age-specific disease incidence, survival, prevalence, and longitudinal study designs, without language restrictions: “(“age-specific” [Title/Abstract] OR “by age” [Title/Abstract]) AND (incidence [Title/Abstract] OR “incidence rate” [Title/Abstract]) AND (survival [Title/Abstract] OR fatality [Title/Abstract] OR mortality [Title/Abstract] OR lethality [Title/Abstract] OR “case fatality” [Title/Abstract] OR prognosis [Title/Abstract]) AND (prevalence [Title/Abstract] OR “disease frequency” [Title/Abstract] OR “disease burden” [Title/Abstract]) AND (change∗ [Title/Abstract] OR trend∗ [Title/Abstract] OR “over time” [Title/Abstract] OR “time trend” [Title/Abstract] OR “temporal trend” [Title/Abstract] OR “temporal change” [Title/Abstract] OR “temporal dynamic∗” [Title/Abstract] OR evol∗ [Title/Abstract] OR “time evolution” [Title/Abstract] OR increase∗ [Title/Abstract] OR decrease∗ [Title/Abstract] OR growth∗ [Title/Abstract] OR decline∗ [Title/Abstract] OR fluctuat∗ [Title/Abstract] OR dynamic∗ [Title/Abstract]) AND (longitudinal [Title/Abstract] OR “cohort study” [Title/Abstract] OR “prospective study” [Title/Abstract] OR “birth cohort∗” [Title/Abstract]) AND (“multiple diseases” [Title/Abstract] OR “various diseases” [Title/Abstract] OR “different diseases” [Title/Abstract] OR “disease comparison” [Title/Abstract] OR “multi-disease” [Title/Abstract] OR “across diseases” [Title/Abstract])” and “(TI = (“age-specific” OR “by age”) OR AB = (“age-specific” OR “by age”)) AND (TI = (incidence OR “incidence rate”) OR AB = (incidence OR “incidence rate”)) AND (TI = (survival OR fatality OR mortality OR lethality OR “case fatality” OR prognosis) OR AB = (survival OR fatality OR mortality OR lethality OR “case fatality” OR prognosis)) AND (TI = (prevalence OR “disease frequency” OR “disease burden”) OR AB = (prevalence OR “disease frequency” OR “disease burden”)) AND (TI = (change∗ OR trend∗ OR “over time” OR “time trend” OR “temporal trend” OR “temporal change” OR “temporal dynamic∗” OR evol∗ OR “time evolution” OR increase∗ OR decrease∗ OR growth∗ OR decline∗ OR fluctuat∗) OR AB = (change∗ OR trend∗ OR “over time” OR “time trend” OR “temporal trend” OR “temporal change” OR “temporal dynamic∗” OR evol∗ OR “time evolution” OR increase∗ OR decrease∗ OR growth∗ OR decline∗ OR fluctuat∗)) AND (TI = (longitudinal OR “cohort study” OR “prospective study” OR “birth cohort∗”) OR AB = (longitudinal OR “cohort study” OR “prospective study” OR “birth cohort∗”)) AND (TI = (“multiple diseases” OR “various diseases” OR “different diseases” OR “disease comparison” OR “multi-disease” OR “across diseases” OR “across disease types”) OR AB = (“multiple diseases” OR “various diseases” OR “different diseases” OR “disease comparison” OR “multi-disease” OR “across diseases” OR “across disease types”))”. We found that previous studies had examined temporal trends in incidence and survival of individual diseases, but no previous study had simultaneously analysed multiple major diseases with distinct aetiologies. Nor had any study systematically summarised and compared age-specific changes in incidence and survival across different diseases and over several decades, including their magnitude of relative improvements at different ages. However, these perspectives are crucial for distinguishing preventive advances occurring before diagnosis (primary prevention) and at or after diagnosis (secondary and tertiary prevention), and for understanding how medical progress alters the disease histories of individuals and the disease landscape of populations.

**Added value of this study**

To the best of our knowledge, this is the first study to systematically compare the long-term impact of medical progress before and after diagnosis across several major diseases with distinct aetiologies and a substantial public health impact (myocardial infarction, stroke, hip fracture, and cancer) in a single analysis. Using population-wide data for 57 birth cohorts spanning three decades, we summarise the changes in incidence and survival for ages 60–90 and compare their effect on disease prevalence at ages 70, 80, and 90. We show that the patterns of change in incidence and survival are disease-specific. For myocardial infarction, stroke, and hip fracture, declines in incidence have exceeded improvements in survival. This reflects substantial progress in preventing severe acute events and results in decreasing prevalence. In contrast, cancer has shown increasing incidence alongside improved survival, leading to rising prevalence. These findings suggest that progress in preventing myocardial infarction, stroke, and hip fracture has been strongest before diagnosis (primary prevention), whereas progress in preventing cancer has been concentrated especially at or after diagnosis (secondary and tertiary prevention). Taken together, our results reflect shifts in the disease landscape of a low-mortality population like Sweden, characterised by an increasingly successful prevention of severe acute events and a growing prevalence of cancer.

**Implications of all the available evidence**

Our results show that an increasing proportion of individuals avoided severe acute events such as myocardial infarction, stroke, and hip fracture, likely by preventing their underlying conditions from escalating. At the same time, a growing proportion of individuals developed cancer, highlighting the impact of earlier and more comprehensive detection. However, those who experienced any of these conditions survived for longer, as improved care extended life beyond the acute phase. The declining prevalence of myocardial infarction, stroke, and hip fracture contrasts with the widely reported rise in multimorbidity. The available evidence thus suggests that the disease burden in ageing populations is shifting rather than decreasing. Severe acute events may be increasingly replaced by chronic progressive conditions that extend over longer periods of life. This is consistent with successful primary prevention, which reduces the occurrence of severe disease manifestations, alongside more effective secondary and tertiary prevention that stabilise underlying chronic conditions. Overall, these developments may imply a reshaping of individual disease histories to longer lives lived with chronic diseases and a greater potential for the accumulation of multiple conditions over the life course.

## Introduction

Medical progress is currently one of the strongest forces changing populations. It influences populations in two fundamental ways: through declining incidence rates (by preventing the onset of diseases) and through improved disease survival (by reducing disease impact and extending life years with the disease). Declining disease incidence largely reflects effective primary prevention before diagnosis, while longer survival with disease mainly results from advances in secondary and tertiary prevention at or after diagnosis. However, it remains unclear which of these two forces—incidence declines or survival improvements—has advanced more, and how their combined effect, which we refer to as the “incidence–survival gap”, has altered the health and composition of populations. In this study, we address this question by examining how the incidence–survival gap of myocardial infarction (MI), stroke, hip fracture, and cancer has developed in Sweden over nearly 60 birth cohorts spanning three decades.

The incidence–survival gap can be seen as a key indicator for monitoring a population's disease burden, as it determines disease prevalence. Disease incidence adds individuals to the pool of those living with a disease, whereas disease survival determines the outflow. The rising prevalence of multimorbidity over time,^1^ alongside increases in both life expectancy and healthy life expectancy,^2^^,^^3^ suggests that improvements in survival may have outpaced declines in incidence for many diseases. However, this issue has not yet been sufficiently addressed. We are not aware of any previous study which has summarised and compared long-term changes in incidence and survival across different diseases, also in relation to prevalence trends. Understanding how the incidence–survival gap has evolved, and how it differs across diseases will provide insights into the processes that have changed the disease landscape of populations.

In this study, we investigate the incidence–survival gap for four major diseases: myocardial infarction, stroke, hip fracture, and cancer. These conditions were chosen as they represent a large share of the overall disease burden in the Swedish population at higher ages. As severe conditions, they have long-lasting negative health consequences. Furthermore, they are among the leading causes of death in Sweden as well as in most European and other high-income countries. From a preventive perspective, the selected conditions are intriguing to study because they can be considered as the final manifestations of pre-existing underlying conditions or the accumulation of unfavourable lifestyle and environmental factors. They differ in their disease progression and causes, yet they also have common and distinct risk factors.^4^ MI, stroke, and hip fracture usually present with an acute onset requiring hospitalisation, while cancer typically follows a multi-stage progression, with a complex and prolonged course. Furthermore, substantial preventive and therapeutic advances have been made in the prevention, diagnosis and treatment of these diseases. Better management of cardiovascular conditions such as hypertension and atrial fibrillation has led to a lower incidence of MI and stroke, and better survival following these events.^5^^,^^6^ For hip fractures, interventions addressing osteoporosis and fall risk aim to reduce incidence, while advances in surgery and postoperative care have improved survival after a hip fracture.^7^ Similarly, cancer incidence and survival have benefited from enhanced screening and diagnostics, as well as major breakthroughs in targeted therapies and immunotherapies.^8^ Not least, MI, stroke, hip fracture, and cancer lend themselves well to study over longer time periods, as they can be robustly identified in either inpatient care and cause of death data (MI, stroke, hip fracture) or in specific disease registers (cancer). Overall, this makes the four diseases ideal candidates for assessing how medical progress has shaped the incidence–survival gap across the disease landscape and over time.

In our analysis, we examine long-term trends in age-specific incidence rates and survival proportions among individuals diagnosed at ages 60–90 and compare their changes over time. Furthermore, we explore how the observed incidence–survival dynamics translated into changing disease prevalence. To the best of our knowledge, this is the first study to systematically compare the long-term impact of medical progress before and after diagnosis across several major diseases with distinct aetiologies and substantial public health impact.

## Methods

### Data

Information was gained from a linkage of several Swedish registers: The population was identified from the Total Population Register and the date and causes of death were retrieved from the Cause of Death Register (CDR). Disease diagnoses were obtained from the National Patient Register (NPR) and the Swedish Cancer Register (SCR). Data covering the entire Swedish population was available from 1 January 1987 until 31 December 2022, except for the SCR, which was available until 31 December 2021. Our analysis includes the cohorts born between 1904 and 1960, which allowed us to incorporate diseases diagnosed between the ages 60–90.

### Identification of incident cases

Cases of MI, stroke, and hip fracture were identified as inpatient stays with the corresponding primary diagnosis in the NPR or as deaths with the respective underlying or contributing cause of death in the CDR. Malignant cancer cases were identified by combining the cancer diagnosis from the SCR with hospital admissions due to a malignant cancer diagnosis according to the NPR and the causes of death (underlying or contributing) from the CDR, whatever comes first. The following ICD codes were used to identify the diagnoses for the diseases of interest: MI: 410 (ICD-9), I21-22 (ICD-10); stroke: 430-434, 436 (ICD-9), I60-I64 (ICD-10); hip fracture: 820 (ICD-9), S72.0–S72.2 (ICD-10); any malignant cancer: 140–209 (ICD-7), 140–209 (ICD-9), C00-C95 (ICD-10). To identify incident cases of MI, stroke, and hip fracture, we continuously applied a seven-year washout period, following the Swedish National Board of Health and Welfare's statistics on the incidence of first MI.^9^ For cancer, we continuously applied a five-year washout period to account for recurring cancer diagnoses in the NPR, considering that patients are typically declared to be in long-term remission if they show no signs of cancer for five years after treatment. All washout periods started in 1987 and information on incident cases could thus be derived from 1994 (MI, stroke, hip fracture) and 1992 (cancer) onwards. Overall, MI, stroke, hip fracture, and cancer can be studied reliably with Swedish administrative health data.^10^^,^^11^

### Calculation of incidence rates, survival proportions, and their changes over time

Sex-, age-, and birth-cohort-specific incidence rates of first events of MI, stroke, hip fracture, and cancer were calculated as the number of cases at each age and birth cohort divided by the person-years at risk. We calculated the one- and five-year survival for incident cases within the initial strata (age at diagnosis, sex, birth cohort) as the proportion of MI, stroke, hip fracture, or cancer cases that were alive one year and five years after the event date. This operationalisation considers the risk of dying from any cause within one and five years.^12^ Notably, all incident cases were considered to be at risk of death, regardless of the register via which they were identified. The relative age-specific changes in incidence rates, one- and five-year survival were assessed by computing age-specific rates of change across birth cohorts (see Supplementary Figure S1 for further details), which were calculated as the natural logarithm of the ratio between two adjacent birth cohorts. We did this for each age from 60 to 90 and derived the average relative cohort-to-cohort change by taking the age-specific mean of all rates of change, respectively. As a supplemental analysis, we determined the extent to which survival improvements differ between the general population and patient populations by comparing the respective one- and five-year survival proportions, as well as the average relative survival improvement over time. The general population consists of the entire Swedish population born between 1904 and 1960 at ages 60–90, containing all individuals living in Sweden irrespective of their health status.

### Calculation of prevalence proportions

We determined the prevalence proportions of MI, stroke, hip fracture, and cancer by sex and birth cohort at the ages 70, 80, and 90. To allow for comparable time trends, the prevalence calculations were based on incident cases that a cohort accumulated always within a 10-year age range (60–69, 70–79, 80–89) and survived until the end of this age range (69, 79, 89). For example, to calculate the prevalence proportion at age 70, we identified all incident cases that occurred between ages 60 and 69 and that were alive at the beginning of age 70. All analyses were conducted using R (version 4.5.1).

### Ethics approval

This study was performed in line with the principles of the Declaration of Helsinki. The study was approved by the regional ethics committee in Stockholm (Dnr 2011/136–31/5, approved 31 May 2011, and amendment 2022-03486-02, approved 6 July 2022). Informed consent is not needed for large-scale register data in Sweden.

### Role of the funding source

The funder had no role in study design, data collection, data analysis, data interpretation, or writing of the report.

## Results

**Fig. 1:**
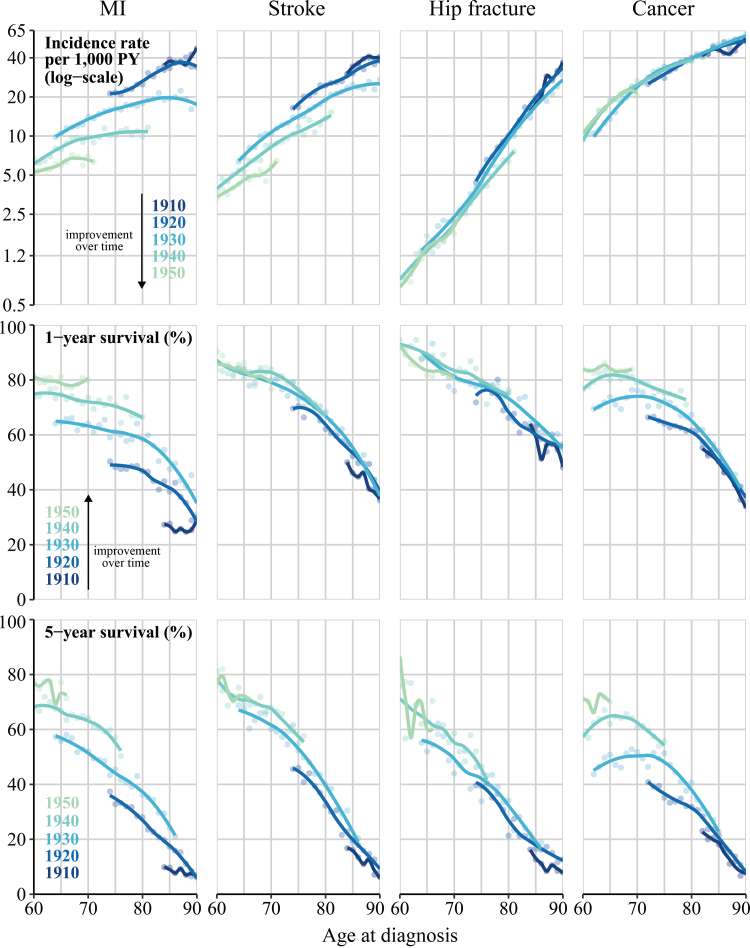
**Age-specific incidence rates, one- and five-year survival proportions of MI, stroke, hip fracture, and cancer at diagnosis ages 60–90, men, birth cohorts 1910, 1920, 1930, 1940, and 1950, Sweden.** Note: survival proportions consider the risk of dying from any cause within one or five years after diagnosis. The solid lines represent the smoothed values (locally estimated scatterplot smoothing (LOESS) with a span of 0.75), while the points show the observed values.

Over the birth cohorts 1904–1960 and the calendar years 1987–2022, we were able to include 5,820,287 individuals in the analysis. Figs. 1 (men) and 2 (women) show the incidence rates (upper panel), one-year (middle panel) and five-year (lower panel) survival proportions for MI, stroke, hip fracture, and cancer at ages 60 to 90 for the selected birth cohorts of 1910, 1920, 1930, 1940, and 1950. These results are based on the calendar years 1994–2022 for MI, stroke, and hip fracture, and 1992–2021 for cancer, depending on age at diagnosis and birth cohort. Incidence rates increased with age, whereas survival proportions declined with age. Men and women exhibited incidence and survival trends that were largely similar. The age-specific incidence rates for MI improved considerably across birth cohorts. For example, the incidence rate for men at age 75 more than halved during a 20-year period, decreasing from 22 (729/33,698) cases per 1000 person-years (PY) in the 1920 cohort to 10 (346/34,133) cases per 1000 PY in the 1940 cohort. Incidence rates of stroke also declined continuously over time. Unlike with MI and stroke, there were only minimal, if any, improvements in hip fracture incidence across cohorts. Cancer incidence increased slightly over time. Among the diseases investigated, cancer had the highest incidence rates at every age.

One-year survival after MI improved considerably across birth cohorts for both men and women. For instance, only 64% (292/455) of men survived for at least one year after having the MI at age 70 in the 1930 birth cohort, while 80% (275/344) of men born in 1950 who had an MI at age 70 survived for at least one year. Compared to MI, the one-year survival of individuals experiencing a stroke or hip fracture increased to a much smaller extent over time. From ages 60 to 70, one-year survival even showed a stagnation (stroke) or a decline (hip fracture) across the compared birth cohorts. With regard to cancer, there were considerable improvements in one-year survival, which were especially pronounced for diagnosis ages 60–80. The patterns of five-year survival were similar to those of one-year survival proportions. The five-year survival for any disease was typically lower than the one-year survival. However, improvements in five-year survival across cohorts were greater than those observed for one-year survival.

**Fig. 2:**
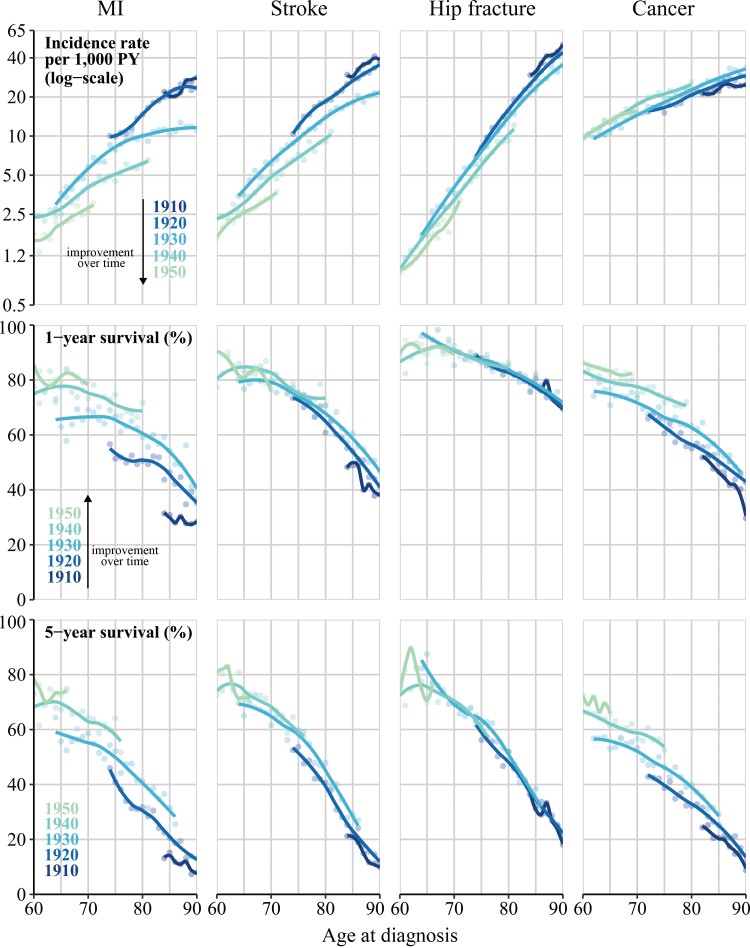
**Age-specific incidence rates, one- and five-year survival proportions of MI, stroke, hip fracture, and cancer at diagnosis ages 60–90, women, birth cohorts 1910, 1920, 1930, 1940, and 1950, Sweden.** Note: survival proportions consider the risk of dying from any cause within one or five years after diagnosis. The solid lines represent the smoothed values (locally estimated scatterplot smoothing (LOESS) with a span of 0.75), while the points show the observed values.

Comparing Figs. 1 and 2 shows some sex-specific differences. Age-specific incidence rates for women were usually lower than for men, except for hip fractures, where the incidence rates were higher for women. In terms of survival, women had a slightly better survival prognosis than men after disease diagnosis, especially after hip fractures. The observed incidence rates, one- and five-year survival proportions of MI, stroke, hip fracture, and cancer in men and women at selected ages of diagnosis and birth cohorts are provided in Supplementary Table S1. Compared to the general Swedish population, women and men who had experienced an MI, stroke, hip fracture, or cancer showed significantly lower survival proportions at any age (Supplementary Figures S2 and S3).

**Fig. 3:**
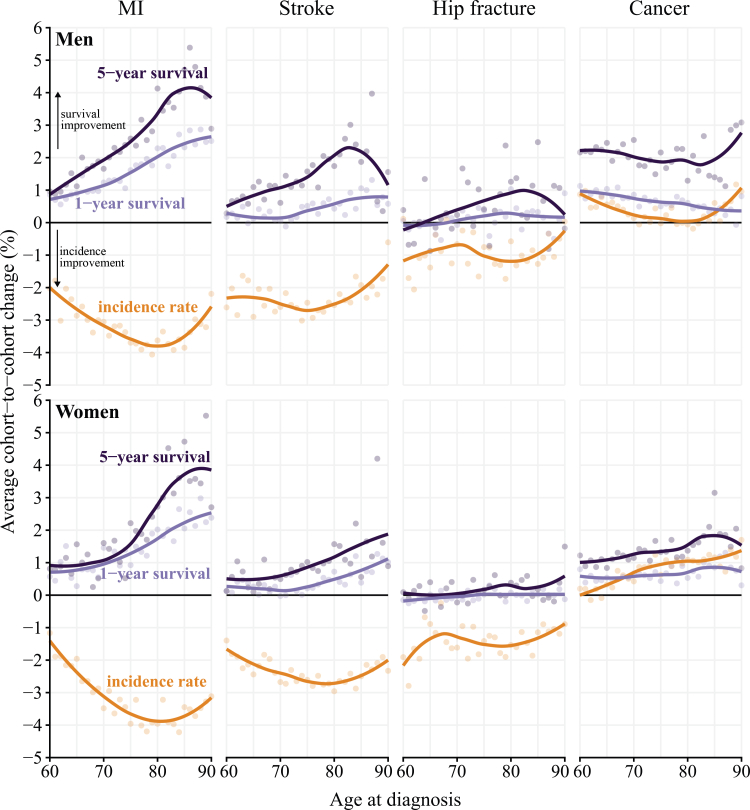
**Average cohort-to-cohort changes (y-axis) in incidence rates, one- and five-year survival proportions for MI, stroke, hip fracture, and cancer at diagnosis ages 60–90 (x-axis) in men and women, Sweden**. Note: survival proportions consider the risk of dying from any cause within one or five years after diagnosis. The solid lines represent the smoothed values (locally estimated scatterplot smoothing (LOESS) with a span of 0.75), while the points show the observed values. The y-axis displays the relative age-specific change between two adjacent birth cohorts averaged over 23–28 birth cohorts, (28 cohorts for incidence, 27 cohorts for one-year survival and 23 cohorts for five-year survival). The changes are based on the years 1994–2022 (MI, stroke, hip fracture) or rather 1992–2021 (cancer).

Fig. 3 illustrates the average relative cohort-to-cohort changes in incidence rates, one-, and five-year survival proportions for MI, stroke, hip fracture, and cancer at ages of 60–90 in men (upper panel) and women (lower panel), calendar years 1994–2022 (MI, stroke, hip fracture) and 1992–2021 (cancer). Women and men presented patterns of incidence and survival changes that were broadly similar. For men, the relative decline in MI incidence was lowest for age 60, with an average decrease of about 2.0% (SD = 7.0%) from one cohort to another, resulting in an absolute decrease from 7 (274/37,411) to 4 (234/54,144) cases per 1000 PY between the 1934 and 1960 birth cohorts. It was largest for age 79, with an average decline of about 3.9% (SD = 7.8%) from one cohort to another, leading to an absolute decrease from 28 (586/20,978) to 9 (291/31,048) cases per 1000 PY between the 1915 and 1942 birth cohorts. One-year survival had the smallest average relative cohort-to-cohort improvement at age 60 (about 0.9% (SD = 3.2%), leading to an absolute increase in one-year survival from 70% (193/274) to 88% (206/234) between the 1934 and 1960 birth cohorts) and the largest at age 90 (about 2.5% (SD = 20.3%), resulting in an absolute rise in one-year survival from 16% (19/118) to 31% (39/126) between the 1904 and 1930 birth cohorts). The declines in incidence were consistently larger than the increases in one-year survival. Compared to five-year survival, the decreases in incidence were larger before age 82, and smaller beyond this age. Similar patterns were observed in stroke. The decreases in incidence were always higher than the improvements in one-year survival, and the improvements in five-year survival approached those in incidence from around age 80 onwards. Compared to MI, both the improvements in stroke incidence and survival were at a lower level. In relation to MI and stroke, hip fractures showed smaller improvements in incidence and survival. There was even a slight reduction in one-year survival at ages 60–70, and in five-year survival at ages 60–65.

Cancer was the only condition with an increase in incidence throughout the age range, particularly at the ages 60–70 and 85–90. At ages 85 and higher, relative increases in incidence were even larger than those in one-year survival. The five-year survival for cancer improved more at all ages in comparison with stroke and hip fracture, and improved more at the ages 60–70 in comparison with MI. Improvements in five-year survival were greater than those in one-year survival for all diseases investigated. When comparing women and men, women exhibited greater declines in hip fracture incidence at all ages, and a more pronounced increase in cancer incidence with age. Improvements in five-year survival for stroke, hip fracture, and cancer were smaller for women. The increases in one- and five-year survival of individuals experiencing an MI, stroke, hip fracture, or cancer exceeded the survival improvements in the general Swedish population (Supplementary Figure S4).

**Fig. 4:**
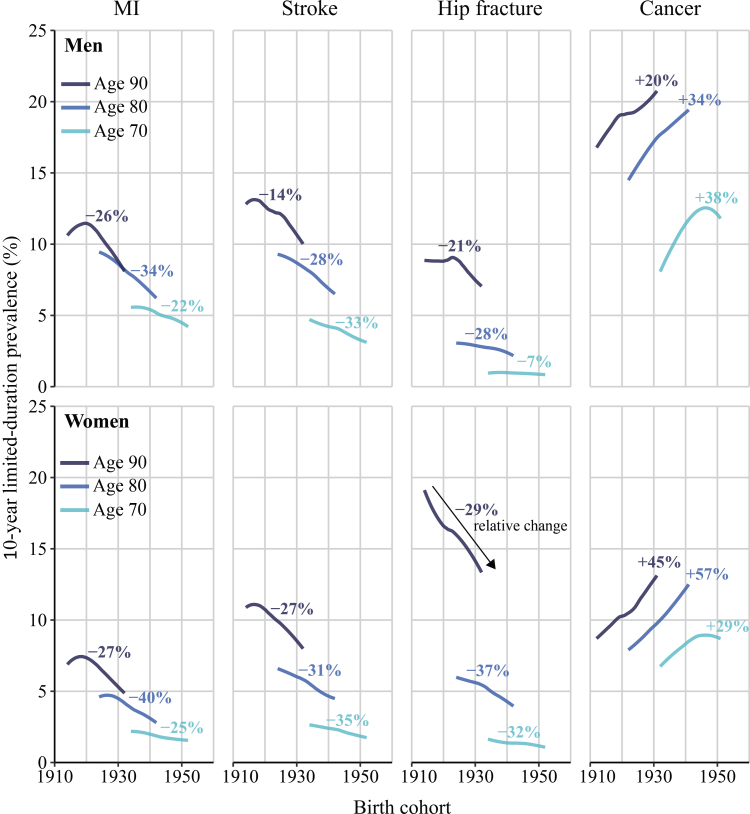
**Ten-year limited-duration prevalence (%) of MI, stroke, hip fracture and cancer in men and women aged 70, 80, and 90, birth cohorts 1912–1952, Sweden.** Note: Age-specific prevalence proportions were based on incident cases occurring within the 60–69, 70–79, and 80–89 age ranges and surviving until the beginning of age 70, 80, and 90, divided by the population alive at the beginning of age 70, 80, and 90, respectively. All results were smoothed using locally estimated scatterplot smoothing (LOESS), with a span of 0.75. For the unsmoothed prevalence figures, see Supplementary Figure S5. Relative changes indicate the relative difference between the first and last observed prevalence at each age.

Fig. 4 shows the prevalence of the four diseases at ages 70, 80, and 90, for men (upper panel) and women (lower panel), across the birth cohorts from 1912–1952, calendar years 1994–2022 (MI, stroke, hip fracture) and 1992–2021 (cancer). The unsmoothed prevalence figures are shown in Supplementary Figure S5 and provided for selected birth cohorts in Supplementary Table S2. The age-specific prevalence of MI, stroke, and hip fracture declined with more recent birth cohorts, whereas it increased for cancer. For men, the prevalence of MI at age 90, for example, was 10.9% (650/5,958) in cohort 1914 and declined to 8.0% (701/8,736) in cohort 1932. This equals to a reduction in prevalence by about 3 percentage points, or a relative decrease by 26%. Compared to MI, the pattern and level of stroke prevalence were largely similar. The prevalence of hip fractures was considerably lower at every age and declined less over time (e.g., among men aged 70, from 0.93% (318/34,177) in cohort 1934 to 0.87% (456/52,472) in cohort 1952, corresponding to a relative change of −7%). Cancer prevalences in men were the highest at every age and showed an increase with more recent cohorts. At age 90, more than one in five men (20.7% (1,800/8,685)) from the 1931 birth cohort were diagnosed with cancer between ages 80 and 89, considering all incident cases in that age range. This is an increase by 3.4 percentage points compared to the 1912 cohort with a cancer prevalence of 17.3% (969/5,595), equivalent to a relative change of 20%. The strongest relative growth was observed for cancer prevalence at age 70 among birth cohorts from 1932 (prevalence of 8.3% (2,916/35,026)) to 1951 (prevalence of 11.5% (5,962/52,010)), corresponding to a relative change of 38%. In comparison to men, the prevalence magnitude for MI, stroke, and cancer was lower among women of all ages. Conversely, hip fractures were more prevalent in women, especially at age 90, where the hip fracture prevalence consistently exceeded even the cancer prevalence. Notably, women experienced greater reductions in the prevalence of MI (e.g., among women aged 80, from 4.5% (1,554/34,676) in cohort 1924 to 2.7% (1,105/41,122) in cohort 1942, reflecting a relative change of −40%), stroke, and hip fracture, and larger relative increases in the prevalence of cancer across birth cohorts (e.g., among women aged 80, from 8.0% (2,832/35,568) in cohort 1922 to 12.5% (4,563/36,609) in cohort 1941, corresponding to a relative change of 57%). Overall, decreasing prevalences were observed for diseases where the average cohort-to-cohort decline in incidence was greater than the increase in survival. This was the case for MI, stroke, and hip fracture, where relative improvements in incidence outpaced those in survival at every age (see Fig. 3). Likewise, larger average improvements in survival than in incidence resulted in increasing prevalences, as evident for cancer.

## Discussion

This study assessed how changes in incidence and survival with four major diseases have evolved in Sweden over recent decades, and how their combined effect—the incidence–survival gap—has changed prevalence. The results show that the progress against MI, stroke, and hip fracture was stronger before diagnosis (primary prevention) than at or after diagnosis (secondary and tertiary prevention), leading to decreasing prevalence. In contrast, progress against cancer was only evident in survival improvements and thus, at or after diagnosis, leading to rising prevalence. This pattern reflects shifts in the disease landscape of a low-mortality population like Sweden, characterised by an increasingly successful prevention of severe acute events and a growing prevalence of cancer, a chronic and progressive disease. However, those who still experience these four severe conditions are surviving for longer, as medical progress extends life beyond the acute phase. Both developments imply longer lives with chronic diseases and may allow individuals to accumulate additional diseases over their remaining life span.

We also find marked differences between improvements in one- and five-year survival, which suggest that progress in these two phases depends on distinct determinants. One-year survival is highly influenced by the acute course of the disease and the effectiveness of the emergency response and immediate treatment.^13^ In contrast, long-term survival is additionally shaped by the selection of individuals who have survived the acute phase and who may possess more favourable health profiles, stronger social support, or better access to continuing care.^14^ This selection has likely amplified the progress in five-year survival. Moreover, as incidence declines and fewer individuals develop these diseases, those who do may be increasingly concentrated among higher-risk groups with more complex health profiles. This shift has possibly reduced the pace of improvements in short-term survival. By contrast, improvements in long-term survival also benefit from chronic disease management and broader population health gains.

The observed incidence and survival trends also reflect that the selection into certain conditions has changed over time. Diagnostics have become more sensitive for MI (with the implementation of (high-sensitivity) troponin assays), stroke (with more CT/MRI scanning for minor symptoms), and cancer (with screening and better imaging). This has resulted in a more comprehensive detection of these conditions, especially for milder and early-stage manifestations. This suggests, on the one hand, that the decline in incidence rates for MI and stroke would have been even more pronounced if the diagnostics had not improved. For cancer, the increases in incidence were so remarkable that they may not only result from improved diagnostics alone.^15^^,^^16^ On the other hand, improved diagnostics may have changed the pool of patients to comprise not only more cases, but also, with everything else staying constant, a rising number of milder cases with a better survival prognosis. This compositional change of patients may have contributed to the observed survival improvements for MI, stroke, and cancer. By contrast, there was little scope for improvements in the diagnostics of hip fracture, which limits effects on incidence and survival trends in hip fracture patients. Nevertheless, the composition of hip fracture patients has also changed. A previous study has reported a higher proportion of hip fracture patients in Sweden, who are already frail at the time of the fracture.^7^ This demonstrates that selection into the event, driven by either better diagnostics or changing risk factor profiles, is a general methodological issue that must be considered when comparing and interpreting changes in disease incidence and survival over a long period. To address this adequately, further research should incorporate more detailed patient histories across healthcare sectors that precede severe disease manifestations.

The disease-specific pattern in the incidence–survival gap indicates changes in underlying risk factors over time. Improvements in blood pressure and lipid control,^17^ the widespread use of preventive medication, and large reductions in smoking and alcohol consumption^18^^,^^19^ have probably played a central role for the declines in incidence for MI and stroke. These achievements have likely counterbalanced the negative health consequences of rising obesity prevalence among Swedish adults since 1995.^20^ The decrease in incidence rates for MI and stroke was less pronounced at ages 81–90 compared to age 80. This is related to the periods of stagnation or slight increase in incidence rates, experienced by the older birth cohorts included in this study when they were aged 81–90.

Cancer illustrates a different incidence–survival gap since incidence increased. Here, the increasing incidence alongside better survival signals the impact of earlier and more comprehensive detection through screening and diagnostic techniques^21^ rather than a deterioration in primary prevention. Like MI and stroke, cancer is a disease with widespread diagnostic innovations that directly influence incidence. As population-based cancer screening programmes in Sweden, screening for cervical cancer (implemented nationwide during 1967–1977) led to remarkable long-term decreases in cervical cancer incidence,^22^^,^^23^ while the screening for breast cancer (nationwide coverage since 1997) resulted in sharply rising breast cancer incidence in the years following its introduction.^15^ In the last decades, participation rates in these major programmes were comparably high and relatively constant. In contrast, screening for colorectal cancer was implemented unevenly across Sweden's geographical regions and was rolled out across the whole country only since 2022. Full coverage of the target age group (60–74) has yet to be accomplished.^21^ A previous study has found a significant net reduction in colorectal cancer incidence in the 60–74 age group following the introduction of screening in Stockholm and Gotland—the only regions in which screening had been in place since 2008.^24^ Overall, population-based cancer screening programmes cannot be expected to have driven the observed incidence trends for all malignant cancers, as they were either already widely or only selectively implemented during the period of the current study and had varying effects on age-specific incidence rates by cancer subtype. Yet roughly one quarter of cancer cases in Sweden remain attributable to modifiable risk factors,^25^ such as smoking, ultraviolet radiation, and obesity. Given that major advances in managing many of these exposures have already been achieved,^21^ progress in reducing the cancer prevalence will depend on continued efforts in primary prevention and further medical innovations. This underscores that changes in the incidence–survival gap result not only from medical advances but also from the disease-specific interplay of risk factors and prevention strategies.

At first glance, the declining prevalences of MI, stroke, and hip fracture seem to contradict the overall rise in multimorbidity over the same period.^1^ However, the declines in prevalence might indicate that the disease burden is shifting, with acute and severe events increasingly being replaced by chronic progressive conditions that extend over longer periods of life (e.g. hypertension, heart failure, osteoporosis). These dynamics probably reflect more effective secondary and tertiary prevention that stabilise underlying chronic diseases and thus reduce the occurrence of severe health manifestations.^26^ In cardiovascular health, improved management of hypertension, diabetes, and atrial fibrillation^6^ has not only prevented acute events but also prolonged life with chronic diseases. Similar trends are evident for hip fractures through increases in osteoporosis medication and improved functional outcomes after hip fracture surgery.^27^^,^^28^ The decreasing incidence of MI, stroke, and hip fracture also makes these conditions less important as competing events for other conditions, potentially contributing to the observed rise in cancer prevalence.

Like other Nordic countries, Sweden has been an early and systematic adopter of preventive innovations, making it a relevant case study. Its universal healthcare system and strong public health infrastructure^29^ have ensured that medical innovations reached large parts of the population in a timely manner. In addition, Sweden has experienced substantial improvements in modifiable risk factors, such as a reduction in smoking, over the last decades.^19^ This combination of accessible healthcare and population-level risk reduction makes Sweden an ideal setting to examine how the incidence–survival gap has evolved during a period of major medical advances and public health interventions. The shift in the disease burden will alter patient profiles and thus potentially imply a reallocation of resources within the healthcare system to meet the evolving needs in certain medical areas, such as oncology. Additionally, the increasing proportion of people surviving severe diseases means a growing demand for rehabilitative care to treat the direct and indirect health consequences of experiencing severe disease episodes. In that regard, approaches of integrated care will become increasingly important for effectively addressing more complex disease trajectories from an interdisciplinary, intersectoral and patient-centred angle. Although differences between countries might persist in how preventive innovations are implemented, how risk factors are distributed, and how contextual factors influence the incidence–survival gap,^30^ Sweden may still represent a vanguard population in terms of the factors determining disease incidence and survival. As knowledge of medical progress is broadly comparable across high-income countries, we expect our findings to be largely generalisable and to serve as a benchmark for understanding trends observed in other low-mortality populations, even if they are at a different stage of development to Sweden.

A key strength of this study is its use of nationwide Swedish register data spanning more than 30 calendar years and almost 60 birth cohorts, which allowed us to capture longitudinal changes in incidence and survival for multiple major diseases in the entire population between ages 60 and 90. Using a cohort perspective and precise diagnosis timing, we provided a detailed assessment of how the incidence–survival gap has evolved and affected disease occurrence in the population over time. Limitations include the focus on selected severe diseases, which do not represent the whole disease panorama, and the aggregation of cancer into a single category, which masks subtype-specific patterns. In addition, the prevalence calculations were based on incident cases accumulating within 10-year age ranges, which allows for comparable trends over time but does not capture a lifelong perspective. Consequently, prevalence levels may be lower compared to lifetime prevalence estimates, especially at higher ages. This may be particularly relevant for the prevalence of cancer, since the steady increase in cancer risk with age becomes notably more pronounced after the age of 50, and our study does not cover incident cancer cases in the 50–59 age range.

In conclusion, monitoring both the inflow and outflow of diseases provides important insights into changes in a populations’ disease burden: The decreases in incidence of MI, stroke, and hip fracture were so substantial that they offset an increase in prevalence that would have occurred due to improved survival. For cancer, rising incidence rates and extended survival have determined its increasingly significant role within the disease landscape. Future research should explore how the incidence–survival gap contributes to multimorbidity and the increases in life expectancy and health life expectancy, as ageing populations might increasingly experience longer lives shaped by complex, chronic disease trajectories.

## Contributors

CRediT: Conceptualisation: AP, KM, ME; Data access: AP, KM, KSM, ME; Data curation: AP, ME; Data verification: AP, KM, ME; Formal Analysis: AP, ME; Investigation: AP, KM, ME; Methodology: AP, ME; Project administration: AP; Resources: KM; Software: AP; Supervision: KM, ME; Validation: AP, ME; Visualisation: AP; Writing—original draft: AP, ME; Writing—review & editing: AP, KM, KSM, ME; All authors read and approved the final version and had final responsibility for the decision to submit the manuscript for publication.

## Data sharing statement

The individual level data underlying this study cannot be shared publicly because of the General Data Protection Regulation in Sweden. Access to the data can be permitted to external researchers after ethical vetting and establishment of a collaboration agreement. Contact the corresponding author for questions about data sharing. All statistical codes and the data points used for the figures can be found under: https://doi.org/10.17605/OSF.IO/FH9M2.

## Declaration of generative AI and AI-assisted technologies in the manuscript preparation process

During the preparation of this work, the authors used DeepL to improve the clarity and style of the manuscript text and ChatGPT (OpenAI) to assist in revising statistical code and exploring academic literature. After using this tool/service, the authors reviewed and edited the content as needed and take full responsibility for the content of the published article.

## Declaration of interests

AP declares funding by the International Max Planck Research School for Population, Health and Data Science (IMPRS-PHDS) for a research stay at Karolinska Institutet. KSM declares having received funding from the Swedish Research Council for Health, Working Life and Welfare (FORTE), the Swedish Research Council (VR), the Swedish Society of Medicine (SLS), a regional funding from CIMED and the Region Stockholm, a professional fee by the Swedish Heart Lung Foundation for a lecture, and payment for lectures from the Swedish Society of Internal Medicine, and the Regional Drug Committee of the Swedish Region Västernorrland. KM declares having received a research grant from the Swedish Research Council for Health, Working Life and Welfare (FORTE) and being a board member in the National Screening Board at the Swedish National Board of Health and Welfare during the past 36 months. ME declares no competing interests.
