# Supplementary material for: Incidence, survival, and prevalence trends for myocardial infarction, stroke, hip fracture, and cancer across Swedish birth cohorts: a population-based register study
Source: Lancet Reg Health Eur. 2026 Jul 21;68:101785. doi: 10.1016/j.lanepe.2026.101785 (PMC13392955; doi:10.1016/j.lanepe.2026.101785)
Supplement: Supplementary Material [file mmc1.docx]

**Supplementary materials:**

**Incidence, survival, and prevalence trends for myocardial infarction, stroke, hip fracture, and cancer across Swedish birth cohorts: a population-based register study**

Table of Contents

[1 Illustration of the setup for deriving changes in incidence and survival over time 2](#_Toc231285059)

[2 Observed values of the outcome measures for selected ages and birth cohorts 3](#_Toc231285060)

[3 Comparison of changes in one- and five-year survival across birth cohorts between the general population and patient populations 6](#_Toc231285061)

[4 Prevalence figures (unsmoothed) 9](#_Toc231285062)

# **1 Illustration of the setup for deriving changes in incidence and survival over time**


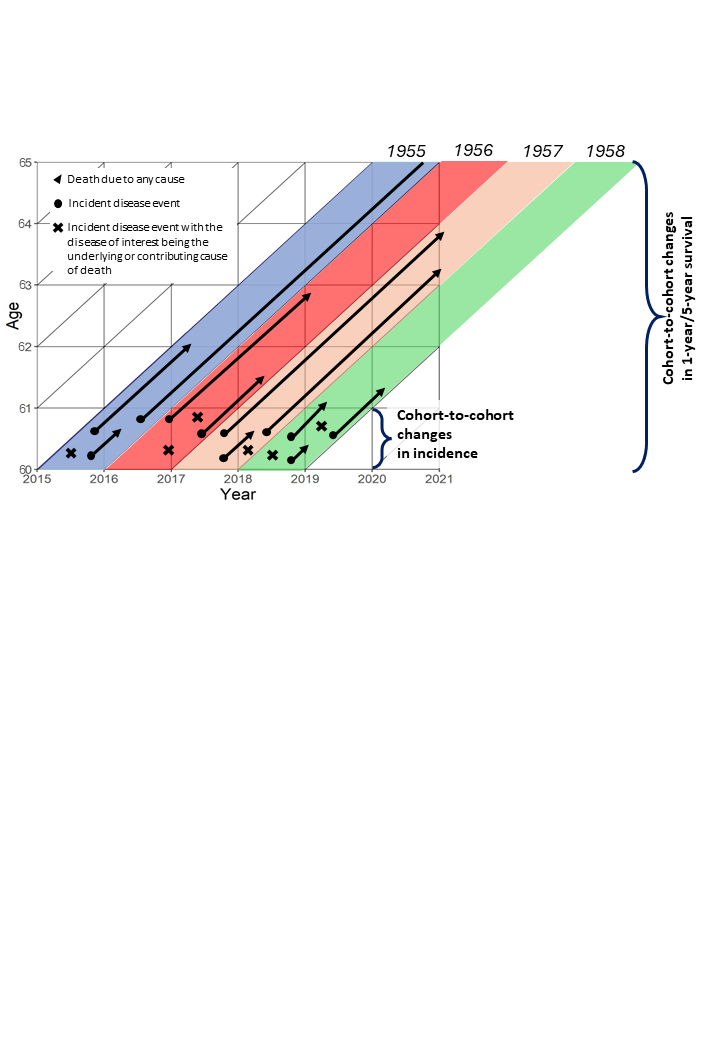


**Figure S1: Calculation of cohort-to-cohort changes in age-specific incidence and one- and five-year survival.**

# **2 Observed values of the outcome measures for selected ages and birth cohorts**

**Table S1: Observed incidence rates, one- and five-year survival proportions of MI, stroke, hip fracture, and cancer in men and women at selected ages of diagnosis and birth cohorts, Sweden.**

|  |  |  | **Men** | | | **Women** | | |
| --- | --- | --- | --- | --- | --- | --- | --- | --- |
| **Condition** | **Age at diagnosis** | **Birth cohort** | **Incidence rate per 1,000 PY** | **1-year survival proportion (%)** | **5-year survival proportion (%)** | **Incidence rate per 1,000 PY** | **1-year survival proportion (%)** | **5-year survival proportion (%)** |
| Myocardial infarction | 60 | 1940 | 6.7 | 73.6 | 67.9 | 2.5 | 77.5 | 73.0 |
|  | 60 | 1950 | 5.2 | 81.7 | 77.2 | 1.8 | 84.8 | 77.8 |
|  | 65 | 1930 | 10.5 | 64.7 | 55.8 | 3.6 | 62.9 | 55.6 |
|  | 65 | 1940 | 7.9 | 74.6 | 66.8 | 2.5 | 77.3 | 67.3 |
|  | 65 | 1950 | 6.0 | 81.3 | 77.1 | 2.1 | 79.6 | 72.6 |
|  | 70 | 1930 | 14.0 | 64.2 | 52.1 | 5.8 | 69.2 | 58.6 |
|  | 70 | 1940 | 9.9 | 73.6 | 64.1 | 4.1 | 73.2 | 64.3 |
|  | 75 | 1920 | 21.6 | 48.1 | 32.8 | 10.0 | 51.3 | 40.8 |
|  | 75 | 1930 | 16.3 | 61.0 | 45.5 | 7.8 | 66.8 | 52.1 |
|  | 75 | 1940 | 10.1 | 70.8 | 56.9 | 4.5 | 74.6 | 64.2 |
|  | 80 | 1920 | 24.9 | 46.8 | 28.0 | 14.6 | 50.5 | 31.8 |
|  | 80 | 1930 | 17.9 | 58.4 | 36.2 | 10.0 | 59.2 | 41.2 |
|  | 85 | 1910 | 38.9 | 27.6 | 9.7 | 20.3 | 30.8 | 15.2 |
|  | 85 | 1920 | 33.2 | 39.4 | 17.3 | 21.8 | 46.4 | 20.6 |
|  | 85 | 1930 | 19.5 | 47.6 | 24.1 | 10.2 | 54.2 | 31.5 |
|  | 90 | 1910 | 47.2 | 29.2 | 6.1 | 28.2 | 28.4 | 8.0 |
|  | 90 | 1920 | 33.8 | 30.0 | 7.0 | 23.5 | 35.2 | 13.1 |
| Stroke | 60 | 1940 | 3.7 | 90.4 | 81.3 | 2.4 | 77.4 | 72.6 |
|  | 60 | 1950 | 3.3 | 83.8 | 75.7 | 1.6 | 90.1 | 81.3 |
|  | 65 | 1930 | 6.8 | 84.2 | 67.6 | 3.5 | 76.7 | 65.1 |
|  | 65 | 1940 | 5.3 | 80.6 | 69.6 | 2.6 | 83.2 | 70.8 |
|  | 65 | 1950 | 4.8 | 86.2 | 72.0 | 2.7 | 80.4 | 73.0 |
|  | 70 | 1930 | 10.4 | 80.1 | 60.1 | 6.1 | 78.9 | 61.6 |
|  | 70 | 1940 | 8.5 | 83.9 | 67.9 | 5.3 | 80.4 | 67.1 |
|  | 75 | 1920 | 16.7 | 70.5 | 46.0 | 11.7 | 73.3 | 53.7 |
|  | 75 | 1930 | 14.0 | 73.2 | 52.3 | 9.4 | 75.5 | 59.7 |
|  | 75 | 1940 | 11.8 | 76.4 | 55.0 | 7.2 | 74.5 | 58.0 |
|  | 80 | 1920 | 24.5 | 62.1 | 32.2 | 18.5 | 60.8 | 40.7 |
|  | 80 | 1930 | 18.8 | 67.9 | 40.0 | 14.0 | 70.6 | 47.9 |
|  | 85 | 1910 | 34.4 | 46.2 | 16.2 | 27.8 | 48.9 | 20.9 |
|  | 85 | 1920 | 30.3 | 52.2 | 19.6 | 27.6 | 54.1 | 22.1 |
|  | 85 | 1930 | 24.8 | 53.5 | 18.7 | 18.1 | 60.6 | 29.0 |
|  | 90 | 1910 | 40.7 | 36.4 | 6.2 | 38.4 | 38.4 | 9.9 |
|  | 90 | 1920 | 37.8 | 37.8 | 8.0 | 36.0 | 41.1 | 11.7 |
| Hip fracture | 60 | 1940 | 0.8 | 94.6 | 75.7 | 0.9 | 90.0 | 75.0 |
|  | 60 | 1950 | 0.7 | 92.5 | 85.0 | 0.9 | 88.7 | 73.6 |
|  | 65 | 1930 | 1.3 | 88.0 | 52.0 | 1.7 | 95.8 | 87.5 |
|  | 65 | 1940 | 1.7 | 89.2 | 55.4 | 1.9 | 89.2 | 71.1 |
|  | 65 | 1950 | 1.5 | 81.7 | 56.1 | 1.4 | 86.1 | 69.6 |
|  | 70 | 1930 | 2.2 | 76.0 | 44.0 | 3.6 | 85.3 | 69.2 |
|  | 70 | 1940 | 2.2 | 81.6 | 58.6 | 3.2 | 92.5 | 70.1 |
|  | 75 | 1920 | 5.3 | 78.2 | 39.4 | 8.2 | 88.0 | 56.1 |
|  | 75 | 1930 | 4.3 | 78.7 | 33.9 | 8.0 | 86.4 | 62.2 |
|  | 75 | 1940 | 4.0 | 79.6 | 46.5 | 6.5 | 86.8 | 61.6 |
|  | 80 | 1920 | 10.1 | 66.2 | 21.3 | 15.7 | 83.3 | 51.9 |
|  | 80 | 1930 | 9.7 | 75.3 | 34.1 | 14.1 | 85.2 | 51.9 |
|  | 85 | 1910 | 18.7 | 63.1 | 12.6 | 30.9 | 78.1 | 31.2 |
|  | 85 | 1920 | 19.8 | 61.1 | 16.4 | 27.9 | 80.5 | 32.3 |
|  | 85 | 1930 | 18.1 | 63.5 | 17.2 | 22.7 | 82.1 | 38.5 |
|  | 90 | 1910 | 37.0 | 48.2 | 7.9 | 50.5 | 69.5 | 18.0 |
|  | 90 | 1920 | 33.6 | 56.6 | 12.7 | 43.5 | 69.7 | 21.3 |

**Table S1 (continued)**

|  |  | |  | **Men** | | | **Women** | | |
| --- | --- | --- | --- | --- | --- | --- | --- | --- | --- |
| **Condition** | **Age at diagnosis** | **Birth cohort** | | **Incidence rate per 1,000 PY** | **1-year survival proportion (%)** | **5-year survival proportion (%)** | **Incidence rate per 1,000 PY** | **1-year survival proportion (%)** | **5-year survival proportion (%)** |
| Cancer | 60 | 1940 | | 9.2 | 76.4 | 58.3 | 9.6 | 82.5 | 66.4 |
|  | 60 | 1950 | | 10.6 | 84.1 | 70.9 | 9.7 | 86.3 | 72.5 |
|  | 65 | 1930 | | 13.5 | 73.8 | 50.8 | 10.7 | 70.8 | 53.0 |
|  | 65 | 1940 | | 19.0 | 80.2 | 62.1 | 13.0 | 76.8 | 61.4 |
|  | 65 | 1950 | | 18.3 | 83.7 | 70.1 | 12.5 | 82.5 | 65.9 |
|  | 70 | 1930 | | 24.3 | 76.2 | 51.8 | 14.8 | 73.5 | 53.3 |
|  | 70 | 1940 | | 26.6 | 78.8 | 60.8 | 17.7 | 75.0 | 56.7 |
|  | 75 | 1920 | | 27.9 | 64.0 | 35.1 | 15.1 | 60.6 | 38.7 |
|  | 75 | 1930 | | 34.2 | 72.3 | 51.3 | 17.1 | 65.7 | 46.2 |
|  | 75 | 1940 | | 34.4 | 75.2 | 55.7 | 19.3 | 71.8 | 52.5 |
|  | 80 | 1920 | | 40.1 | 61.6 | 31.8 | 21.0 | 55.0 | 32.2 |
|  | 80 | 1930 | | 38.2 | 67.4 | 40.6 | 22.8 | 63.4 | 40.0 |
|  | 85 | 1910 | | 45.9 | 50.4 | 18.1 | 22.3 | 46.6 | 20.6 |
|  | 85 | 1920 | | 47.4 | 50.1 | 21.5 | 25.7 | 49.6 | 25.1 |
|  | 85 | 1930 | | 50.6 | 54.0 | 22.8 | 27.2 | 53.5 | 29.1 |
|  | 90 | 1910 | | 54.4 | 33.9 | 8.2 | 24.9 | 29.7 | 8.8 |
|  | 90 | 1920 | | 53.4 | 36.7 | 7.8 | 28.7 | 42.6 | 11.9 |

Note: survival proportions consider the risk of dying from any cause within one or five years after diagnosis. PY = person-years.

**Table S2: Observed 10-year limited-duration prevalence (%) of MI, stroke, hip fracture, and cancer in men and women aged 70, 80, and 90 for selected birth cohorts, Sweden.**

|  |  |  | | **10-year limited-duration prevalence (%)** | |
| --- | --- | --- | --- | --- | --- |
| **Condition** | **Age** | | **Birth cohort** | **Men** | **Women** |
| Myocardial infarction | 70 | | 1935 | 5.7 | 2.4 |
|  | 70 | | 1940 | 5.4 | 2.0 |
|  | 70 | | 1945 | 5.0 | 1.7 |
|  | 70 | | 1950 | 4.5 | 1.6 |
|  | 80 | | 1925 | 9.4 | 4.8 |
|  | 80 | | 1930 | 8.5 | 4.6 |
|  | 80 | | 1935 | 7.5 | 3.7 |
|  | 80 | | 1940 | 6.8 | 3.2 |
|  | 90 | | 1915 | 10.7 | 7.2 |
|  | 90 | | 1920 | 11.5 | 7.5 |
|  | 90 | | 1925 | 9.9 | 6.8 |
|  | 90 | | 1930 | 9.2 | 5.4 |
| Stroke | 70 | | 1935 | 4.6 | 2.6 |
|  | 70 | | 1940 | 4.3 | 2.4 |
|  | 70 | | 1945 | 3.9 | 2.2 |
|  | 70 | | 1950 | 3.3 | 1.9 |
|  | 80 | | 1925 | 9.3 | 6.5 |
|  | 80 | | 1930 | 8.8 | 5.9 |
|  | 80 | | 1935 | 8.0 | 5.5 |
|  | 80 | | 1940 | 6.9 | 4.6 |
|  | 90 | | 1915 | 13.7 | 11.0 |
|  | 90 | | 1920 | 12.8 | 10.5 |
|  | 90 | | 1925 | 12.2 | 10.2 |
|  | 90 | | 1930 | 10.2 | 8.4 |
| Hip fracture | 70 | | 1935 | 1.0 | 1.6 |
|  | 70 | | 1940 | 0.9 | 1.5 |
|  | 70 | | 1945 | 1.0 | 1.4 |
|  | 70 | | 1950 | 0.9 | 1.1 |
|  | 80 | | 1925 | 3.2 | 6.0 |
|  | 80 | | 1930 | 2.9 | 5.7 |
|  | 80 | | 1935 | 2.8 | 5.2 |
|  | 80 | | 1940 | 2.5 | 4.4 |
|  | 90 | | 1915 | 8.0 | 18.9 |
|  | 90 | | 1920 | 8.9 | 16.5 |
|  | 90 | | 1925 | 9.1 | 16.0 |
|  | 90 | | 1930 | 7.5 | 14.0 |
| Cancer | 70 | | 1935 | 9.7 | 7.6 |
|  | 70 | | 1940 | 11.3 | 8.4 |
|  | 70 | | 1945 | 12.4 | 8.9 |
|  | 70 | | 1950 | 12.4 | 8.9 |
|  | 80 | | 1925 | 15.5 | 8.5 |
|  | 80 | | 1930 | 17.8 | 9.6 |
|  | 80 | | 1935 | 18.3 | 10.7 |
|  | 80 | | 1940 | 19.4 | 12.3 |
|  | 90 | | 1915 | 17.3 | 8.8 |
|  | 90 | | 1920 | 18.8 | 10.7 |
|  | 90 | | 1925 | 19.4 | 10.9 |
|  | 90 | | 1930 | 20.4 | 12.6 |

Note: age-specific prevalence proportions were based on incident cases occurring within the 60-69, 70-79, 80-89 age ranges and surviving until the beginning of age 70, 80, 90, divided by the population alive at the beginning of age 70, 80, 90, respectively.

# **3 Comparison of changes in one- and five-year survival across birth cohorts between the general population and patient populations**


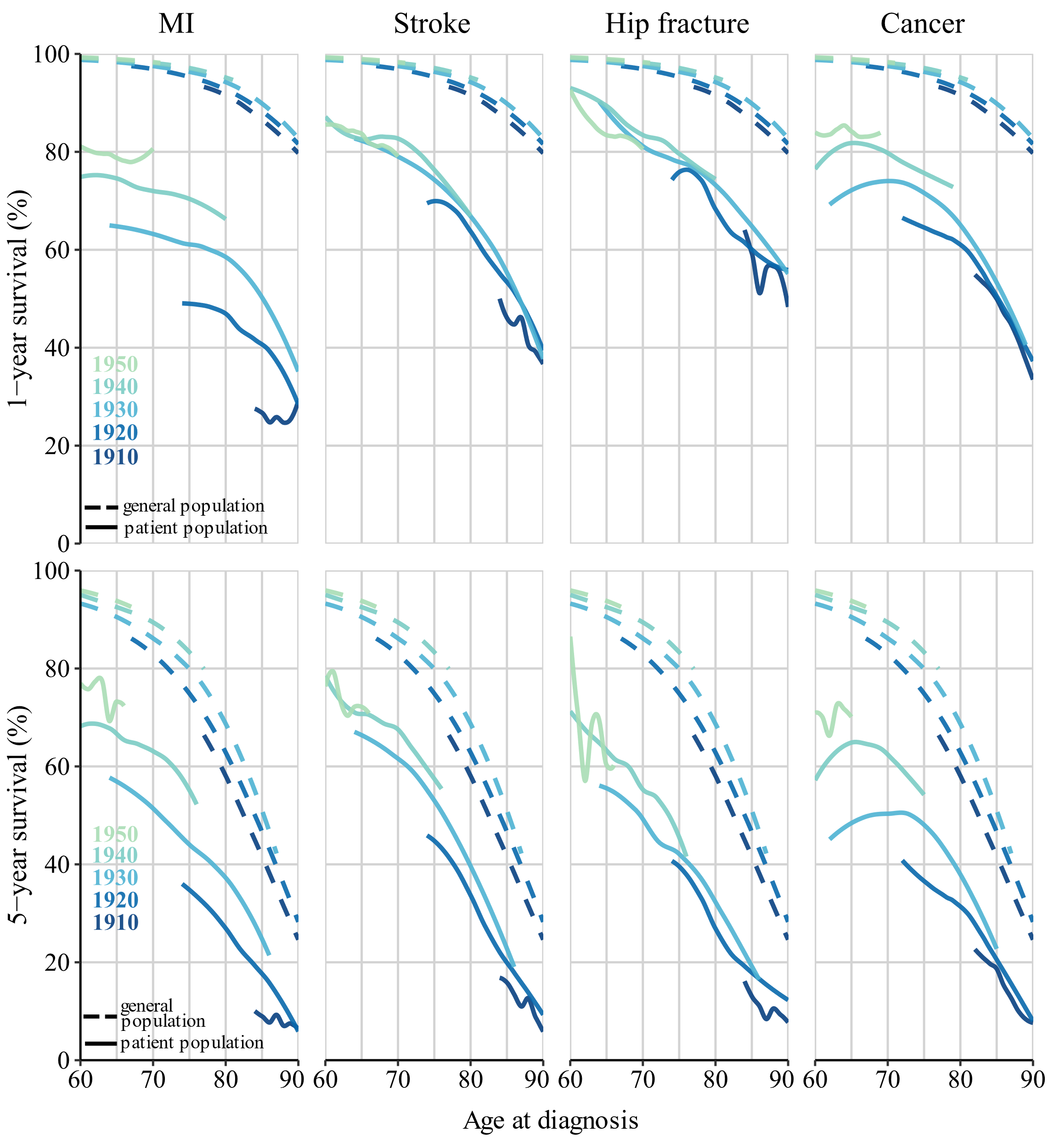


**Figure S2: One- and five-year survival proportions of MI, stroke, hip fracture, and cancer at diagnosis ages of 60 to 90, men, birth cohorts 1910, 1920, 1930, 1940, and 1950, Sweden.** Note: survival proportions consider the risk of dying from any cause within one or five years after diagnosis. The lines represent the smoothed values (locally estimated scatterplot smoothing (LOESS) with a span of 0.75).


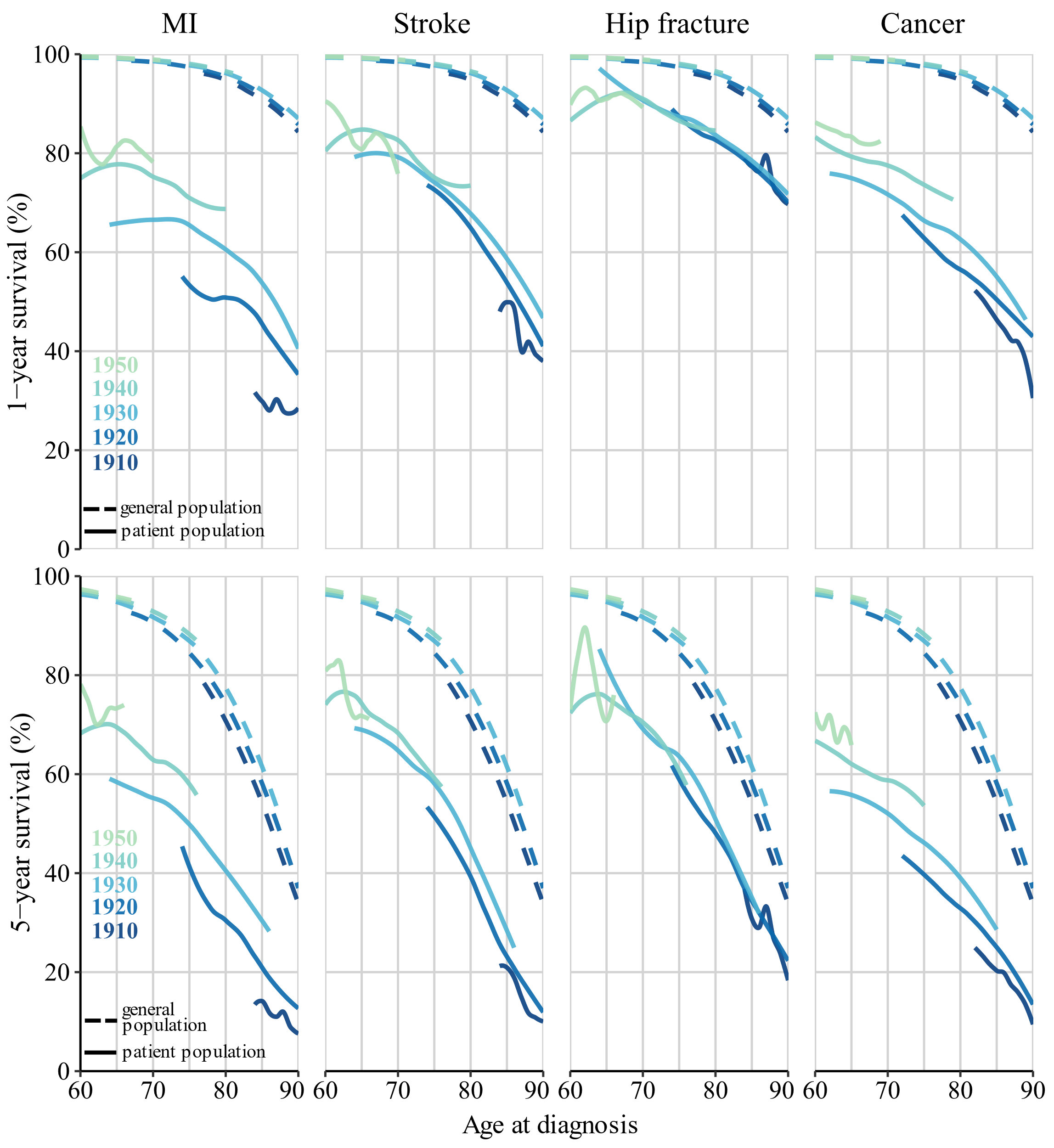


**Figure S3: One- and five-year survival proportions of MI, stroke, hip fracture, and cancer at diagnosis ages of 60 to 90, women, birth cohorts 1910, 1920, 1930, 1940, and 1950, Sweden.** Note: survival proportions consider the risk of dying from any cause within one or five years after diagnosis. The lines represent the smoothed values (locally estimated scatterplot smoothing (LOESS) with a span of 0.75).


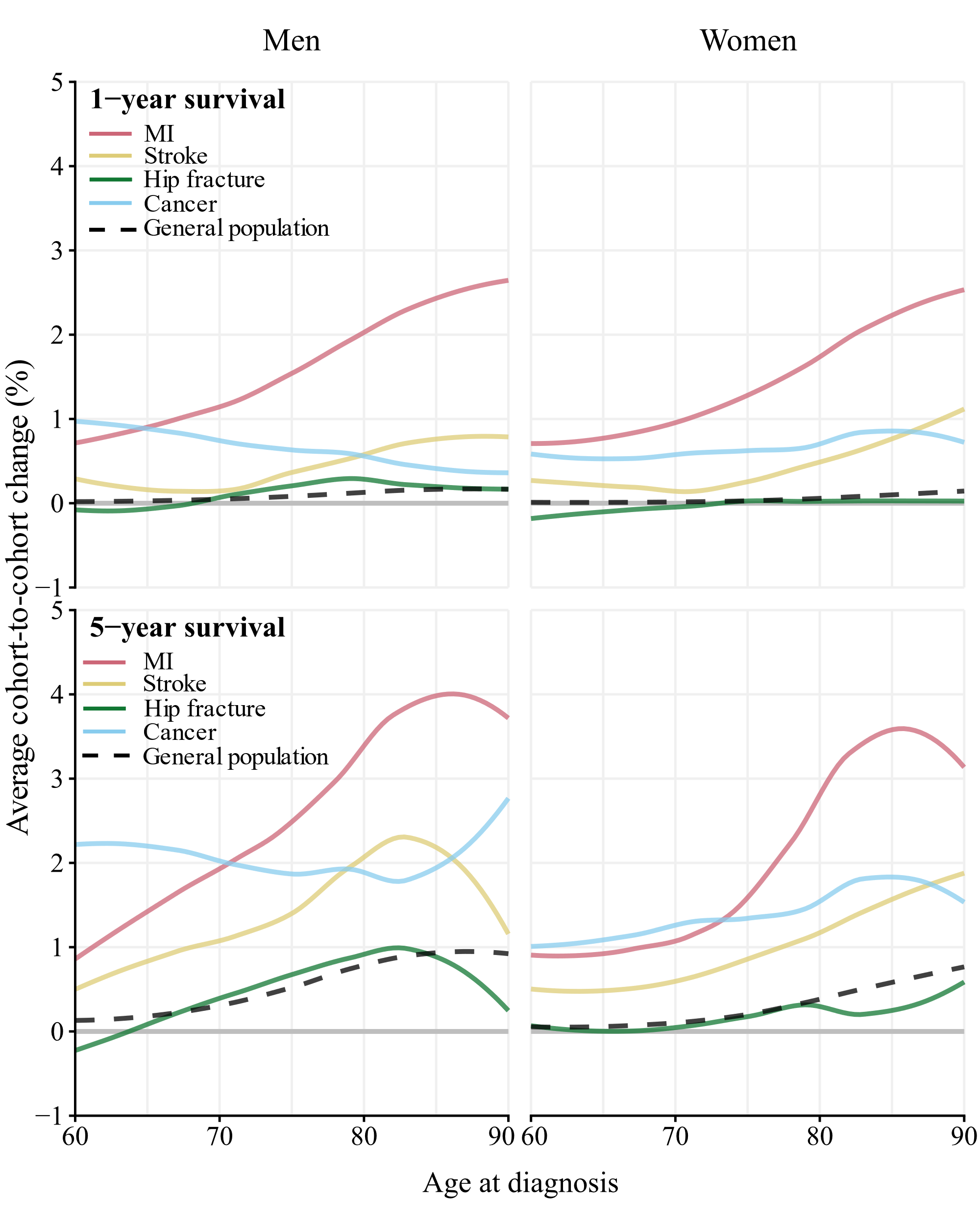


**Figure S4: Average cohort-to-cohort changes (y-axis) in one- and five-year survival proportions for MI, stroke, hip fracture, and cancer at diagnosis ages of 60 to 90 and for the general population at ages 60 to 90 (x-axis) in men and women, Sweden.** Note: survival proportions consider the risk of dying from any cause within one or five years after diagnosis. The solid lines represent the smoothed values (locally estimated scatterplot smoothing (LOESS) with a span of 0.75). The y-axis displays the relative age-specific change between two adjacent birth cohorts averaged over 27 and 23 cohorts for one- and five-year survival, respectively. The changes are based on the years 1994 to 2022 (MI, stroke, hip fracture, general population) or rather 1992 to 2021 (cancer).

# **4 Prevalence figures (unsmoothed)**


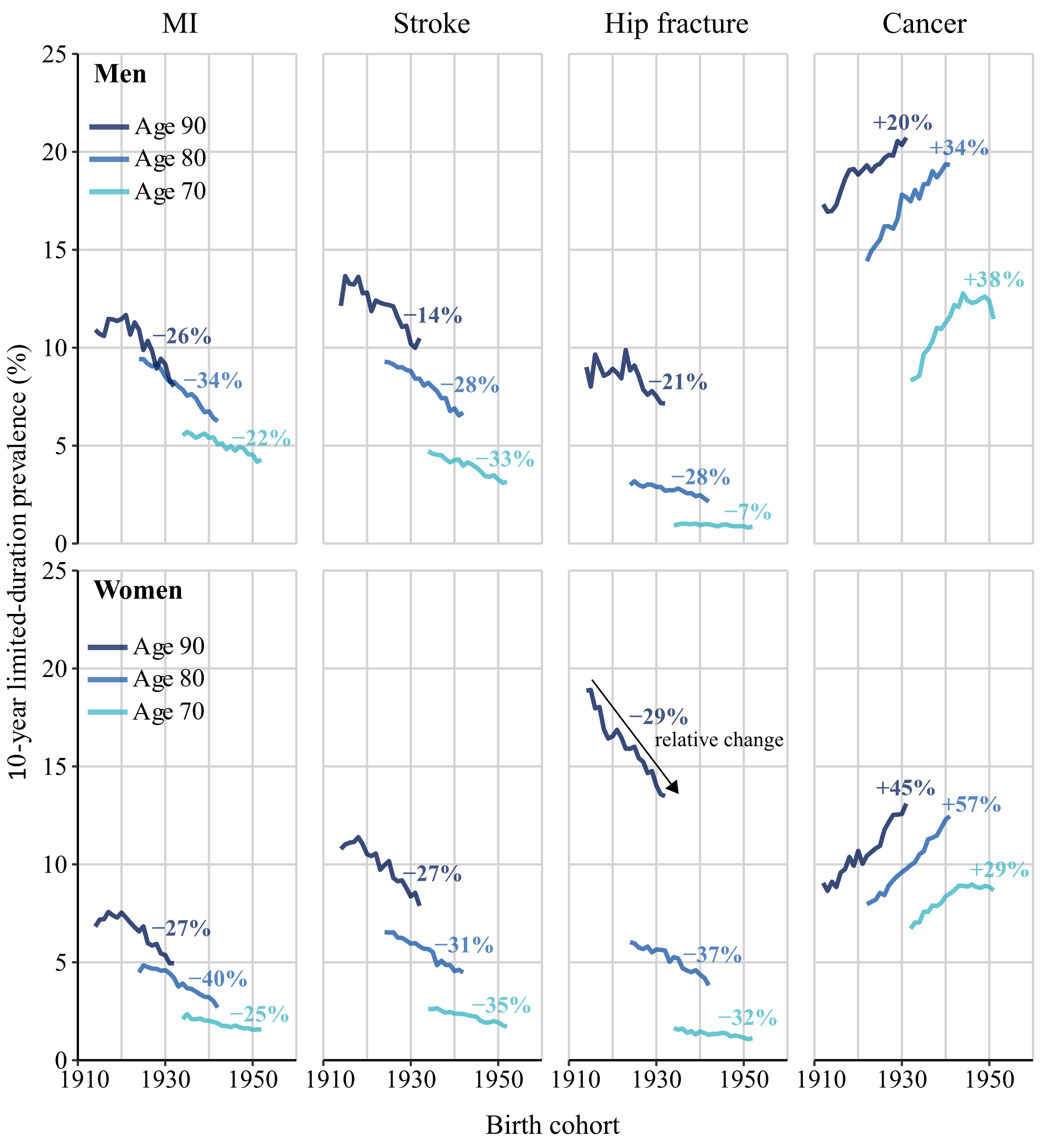


**Figure S5: 10-year limited-duration prevalence (%) of MI, stroke, hip fracture and cancer in men and women aged 70, 80 and 90, birth cohorts 1912 to 1952, Sweden.** Note: Age-specific prevalence proportions were based on incident cases occurring within the 60-69, 70-79, 80-89 age ranges and surviving until the beginning of age 70, 80, 90, divided by the population alive at the beginning of age 70, 80, 90, respectively. Relative changes indicate the relative difference between the first and last observed prevalence at each age.
